# Discovery of a Novel Ubenimex Derivative as a First-in-Class Dual CD13/Proteasome Inhibitor for the Treatment of Cancer

**DOI:** 10.3390/molecules28176343

**Published:** 2023-08-30

**Authors:** Jian Zhang, Simin Sun, Jinyu Liu, Liang Zhang, Di Guo, Naixin Zhang, Jun Zhao, Dexin Kong, Tongqiang Xu, Xuejian Wang, Wenfang Xu, Xiaoyang Li, Yuqi Jiang

**Affiliations:** 1 College of Pharmacy, Weifang Medical University, Weifang 261053, China; zhangjian_3323@163.com (J.Z.);; 2 Key Laboratory of Marine Drugs, Chinese Ministry of Education, School of Medicine and Pharmacy, Ocean University of China, 5 Yushan Road, Qingdao 266003, Chinalixiaoyang@ouc.edu.cn (X.L.); 3 Tianjin Key Laboratory on Technologies Enabling Development of Clinical Therapeutics and Diagnostics, School of Pharmacy, Tianjin Medical University, Tianjin 300070, China; 4 Marine Biomedical Research Institute of Qingdao, Qingdao 266071, China; 5 Laboratory for Marine Drugs and Bioproducts, Qingdao National Laboratory for Marine Science and Technology, Qingdao 266237, China

**Keywords:** CD13 inhibitor, proteasome inhibitor, anti-cancer, anti-metastasis, multiple myeloma

## Abstract

The CD13 inhibitor ubenimex is used as an adjuvant drug with chemotherapy for the treatment of cancer due to its function as an immunoenhancer, but it has limitations in its cytotoxic efficacy. The proteasome inhibitor ixazomib is a landmark drug in the treatment of multiple myeloma with a high anti-cancer activity. Herein, we conjugated the pharmacophore of ubenimex and the boric acid of ixazomib to obtain a dual CD13 and proteasome inhibitor **7** (**BC-05**). **BC-05** exhibited potent inhibitory activity on both human CD13 (IC_50_ = 0.13 μM) and the 20S proteasome (IC_50_ = 1.39 μM). Although **BC-05** displayed lower anti-proliferative activity than that of ixazomib in vitro, an advantage was established in the in vivo anti-cancer efficacy and prolongation of survival time, which may be due to its anti-metastatic and immune-stimulating activity. A pharmacokinetic study revealed that **BC-05** is a potentially orally active agent with an F% value of 24.9%. Moreover, **BC-05** showed more favorable safety profiles than those of ixazomib in preliminary toxicity studies. Overall, the results indicate that **BC-05** is a promising drug candidate for the treatment of multiple myeloma.

## 1. Introduction

CD13/aminopeptide N (APN, EC 3.4.11.2) is a 150 kDa zinc-dependent type II metalloprotease that is expressed on various tissues and cell types, particularly on the membranes of tumor cells, such as multiple myeloma, liver cancer, melanoma, ovarian cancer, prostate cancer, colon cancer, pancreatic cancer, breast cancer, and lung cancer cells [1,2,3]. CD13 is considered a “moonlighting ectoenzyme” due to its multiple functions [4]. As it is a proteolytic enzyme, the substrates degraded by CD13 include the intercellular matrix, angiotensin III, and a variety of immune-related active substances, such as interleukin-2, -6, and -8 [4,5]. Therefore, CD13 is involved in tumor invasion and metastasis [6,7], angiogenesis [8], inflammation, and immune regulation [4,9]. CD13’s function in mediating endocytosis makes it an ideal target in the development of antibody-conjugated drugs (ADCs). Zapata et al. reported the first ADC drug, MI130110, bearing a CD13 monoclonal antibody, and it showed significant anti-tumor activity [10]. More recently, it was reported that CD13 might be a biomarker of human hepatoma stem cells and could potentially serve as a potential therapeutic target in hepatocellular carcinoma [11]. CD13-positive cells are always resistant to chemotherapy anti-cancer drugs in vitro and in vivo. Additionally, CD13 has been shown to mediate angiogenesis in the tumor microenvironment [12,13]. As a result, high expression of CD13 is closely related to the poor prognosis and patient survival for various tumors, such as multiple myeloma, liver cancer, pancreatic cancer, and so on [14,15,16].

**Figure 1:**
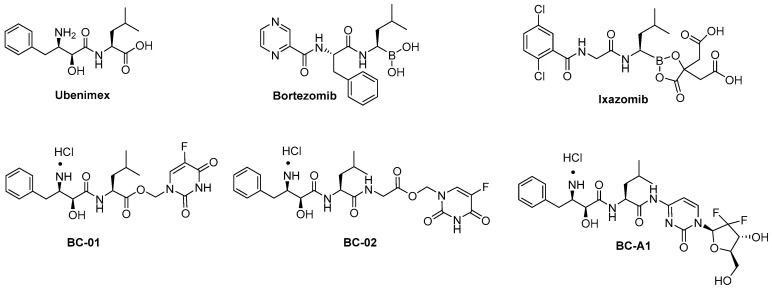
CD13 inhibitors and proteasome inhibitors.

Ubenimex, the only marketed CD13 inhibitor, is used as an adjuvant drug with chemotherapy for the treatment of cancer due to its function as an immunoenhancer (Figure 1) [17,18]. Ubenimex effectively enhances the function of T and B lymphocytes and improves the activity of natural killer (NK) cells. The anti-metastatic and anti-angiogenetic effects of ubenimex in vitro and in vivo have been reported [19,20]. Moreover, ubenimex synergistically enhances the anti-tumor effects of cytotoxic anti-tumor agents, such as 5-fluorouracil (5FU), gemcitabine (GEM), cisplatin (CDDP), and doxorubicin (DXR) [11,21,22,23]. However, the anti-proliferation activity of ubenimex is not apparent even at high concentrations up to 1 mM [24,25]. This disadvantage of ubenimex widely limits its clinical application.

With the approval of bortezomib and ixazomib, proteasome inhibitors have become landmark drugs in the treatment of multiple myeloma (Figure 1) [26]. Ixazomib (MLN9708) is the first FDA-approved orally bioavailable boric acid proteasome inhibitor for the treatment of multiple myeloma. Ixazomib displayed remarkable anti-proliferation efficacy against a panel of tumor cells with a low-nanomole IC_50_ value in vitro [27]. However, ixazomib has a narrow therapeutic window of ixazomib, which can lead to serious side effects such as hematological toxicity, peripheral neuropathy, and abnormal liver function at therapeutical concentrations [28]. Thus, new proteasome inhibitors with higher tolerability are urgently required.

**Figure 2:**
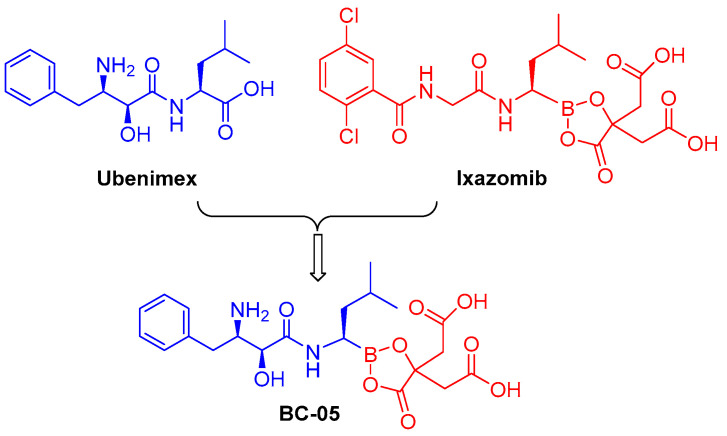
Design strategy, the chemical structure of compound **BC-05**.

The treatment of cancer is a complex process, and it is difficult to achieve ideal treatment results with a single-target inhibitor. The development of multi-target anti-cancer agents has been regarded as an effective strategy for drug discovery in recent years [29]. In our previous study, we designed ubenimex-5FU conjugates (**BC-01**, **BC-02**) and an ubenimex-GEM hybrid (**BC-A1**) as multi-functional agents (Figure 1) [25,30,31,32]. Among these compounds, **BC-02** displayed significant in vitro and in vivo anti-tumor activity [25]. Our studies provide evidence of the concept of covalently linking an appropriate anti-tumor agent to the carboxyl group of ubenimex to maintain potent CD13 inhibitory activity and significantly enhance the anti-cancer efficacy in vitro and in vivo. Based on the advancements and limitations of the CD13 inhibitor ubenimex and proteasome inhibitors ixazomib, we introduced a borate citrate fragment of ixazomib to the carboxyl tail of ubenimex to obtain a CD13/proteasome multi-target inhibitor **BC-05** (Figure 2). We expect to achieve the following goals: (1) the multi-target conjugate **BC-05** will keep increasing the CD13 inhibitory activity of ubenimex; (2) **BC-05** will display inhibitory activity of the proteasome, which increases the anti-proliferation efficacy of the CD13 inhibitor; (3) in addition to the cytotoxicity, **BC-05** will establish better anti-metastasis and immune-stimulating efficacy due to the inhibition of CD13, which may be beneficial to its in vivo anti-tumor activity. The anti-cancer efficacy of **BC-05** was evaluated using various anti-cancer models both in vitro and in vivo. **BC-05** exhibits dual inhibitory activity of CD13 and 20S proteasome and displays higher in vivo anti-tumor activities compared to ubenimex and ixazomib. Furthermore, **BC-05** shows a better safety profile than ixazomib in the in vivo anti-tumor experiments and acute toxicity assessment.

## 2. Results

### 2.1. Chemistry

**Scheme 1:**
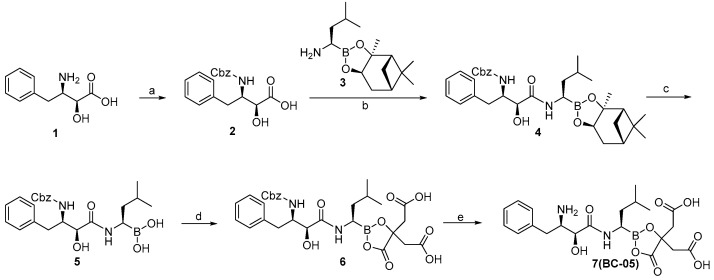
Synthesis of compound **BC-05** *^a^*. *^a^* Reagents and conditions: (a) Cbz-Cl, K_2_CO_3_, THF/H_2_O; (b) EDCI, HOBt, DMF; (c) isobutaneboronic acid, 2N HCl, methanol/hexane; (d) citric acid, EtOAc, rt; (e) Pd/C, H_2_, methanol.

**BC-05** (compound **7**) was synthesized following the procedures shown in Scheme 1. Compound **1** was protected by benzyl chloroformate (Cbz-Cl) to obtain **2**, which was condensed with **3** to obtain the intermediate **4**. The protective group was removed to obtain boric acid **5**, which was further condensed with citric acid to obtain ester **6**. Cleavage of the Cbz protecting group with Pd/C afforded the target product **7** (**BC-05**).

### 2.2. Biological Evaluation of Compound **BC-05**

#### 2.2.1. **BC-05** Is a CD13 and 20S Proteasome Dual Inhibitor

**BC-05** was evaluated for its ability to inhibit CD13 and 20S proteasome. Firstly, both porcine and human CD13 enzymes were used to evaluate the effect of **BC-05** in vitro. Ubenimex and ixazomib were used as positive controls. The data are shown in Table 1. The IC_50_ values of compound **BC-05** toward porcine and human CD13 are 0.65 and 0.13 μM, respectively, which are 7.5 and 15.6 times lower than those of ubenimex. CD13 inhibitory activity of ubenimex has significantly improved after introducing borate citrate. Ixazomib establishes no obvious inhibitory activity against CD13 at a concentration of up to 20 μM. Next, the 20S proteasomal inhibitory activity of the **BC-05** was tested. **BC-05** inhibits chymotrypsin-like (CT-L) activity of the 20S proteasome with IC_50_ of 1.39 μM and the IC_50_ of ixazomib is 0.0024 μM. In general, **BC-05** exhibits potent inhibitory activity against CD13 and modest activity toward 20S proteasome. As we know, proteasome inhibition establishes cytotoxicity on both tumor cells and normal cells, which is the potential reason that highly potent proteasome inhibitors such as ixazomib always display serious target-related toxicity. Therefore, moderate inhibition toward proteasome may be a reasonable choice to balance tolerability and efficacy. 

#### 2.2.2. Docking Study of **BC-05** with CD13 and 20S Proteasome

**Figure 3:**
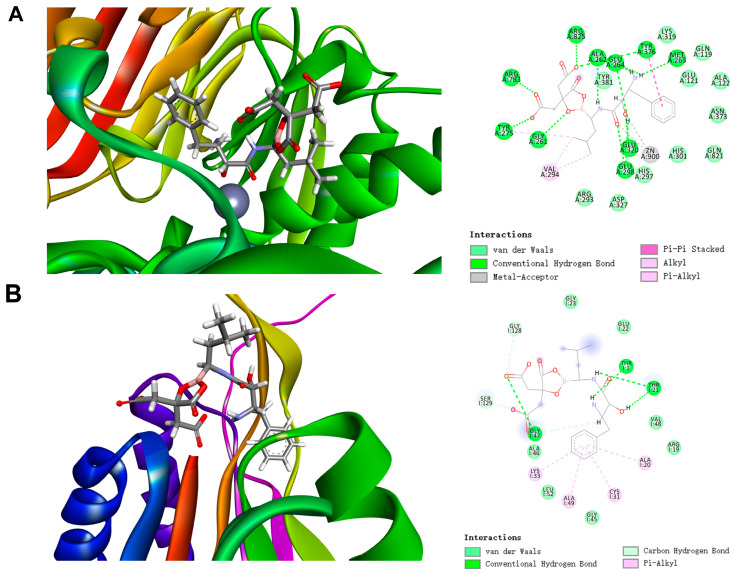
Molecular docking of **BC-05** with the catalytic sites of CD13 (PDB: 2DQM) (**A**) and 20S proteasome (PDB: 7LXT) (**B**) in silico. The molecular docking process was performed using Glide (Schrodinger Inc., supported by the Shanghai Institute of Materia Medica Chinese Academy of Sciences).

To investigate the interaction of **BC-05** with CD13 and 20S proteasome, molecular docking was conducted to predict the binding modes of **BC-05** in the catalytic sites of CD13 (PDB: 2DQM) and 20S proteasome (PDB: 7LXT). Hydrogen and hydrophobic interactions make contributions to the bindings of **BC-05** to the catalytic sites of both CD13 (Figure 3A) and 20S proteasome (Figure 3B). As shown in Figure 3A, the citric acid group of **BC-05** can form multiple hydrogen interactions with surrounding residues such as Ala262, TYR275, Arg783, and Arg825. The amino group has hydrogen interactions with Ala121, Met263, and Glu264. The hydroxyl group has hydrogen interactions with Glu264 and Glu320. One oxygen atom in the borate ester was revealed to form hydrogen interaction with Gly261. There is also hydrogen interaction between the carbonyl group and the surrounding Glu298. Residue Tyr376 plays an important role in the hydrophobic interactions between CD13 and **BC-05**. The hydroxyl group and the carbonyl group of **BC-05** can chelate with the catalytic zinc ion. As revealed in Figure 3B, the citric acid group can also form multiple hydrogen interactions with surrounding residues such as Gly47, Gly128, and Ser129. Both the hydroxyl group and the amide group have hydrogen interactions with Thr21. The amino group has hydrogen interactions with Thr1. Residue Ala20, Cys31, Lys33, and Ala49 play an important role in the hydrophobic interactions between the 20S proteasome and **BC-05**.

#### 2.2.3. **BC-05** Displayed Potent Anti-Proliferative Activity toward Cancer Cell Lines

Ubenimex displays very weak proliferation inhibitory activity in a panel of tumor cell lines (Table 2). The modest inhibition against 20S proteasome may effectively increase **BC-05**’s anti-proliferative activity toward tumor cells. **BC-05** was further evaluated for the anti-proliferative activity using 15 solid or non-solid tumor cell lines. As shown in Table 2, **BC-05** shows moderate inhibitory activity toward these cell lines with micromolar IC_50_ values. Compared with solid tumor cells, **BC-05** shows better inhibitory activity on leukemia and multiple myeloma cells, especially on cell line MM.1S (IC_50_: 1.53 μM). These results demonstrate that the replacement of the carboxylic acid of ubenimex with boric acid effectively increases the anti-proliferative activity. However, the anti-proliferative activity of **BC-05** is weaker than that of ixazomib, which is consistent with the inhibitory activity on the 20S proteasome. In addition, the cytotoxicity of **BC-05** against normal human embryonic kidney cell HEK293 and human hepatocyte HL7702 is much lower than those of ixazomib, revealing potential higher tolerability compared to ixazomib.

#### 2.2.4. **BC-05** Induced Cancer Cell Apoptosis and Cell Cycle Arrest

**Figure 4:**
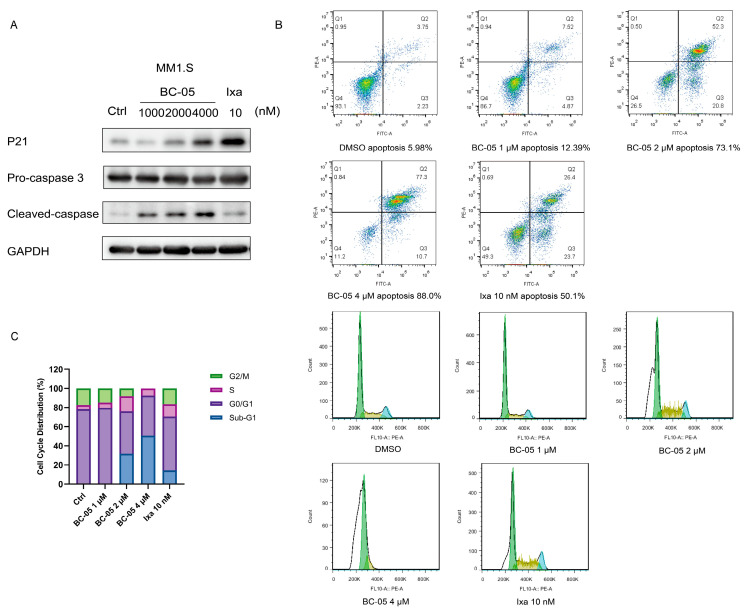
(**A**) Treatment of **BC-05** (1, 2, and 4 µM) and ixazomib (Ixa, 10 nM) in MM1S cell line for 24 h; (**B**) cell apoptosis analysis of **BC-05** (1, 2, and 4 µM) and ixazomib (10 nM) in MM1.S cell line for 48 h; (**C**) cell cycle analysis of **BC-05** (1, 2, and 4 µM) and ixazomib (10 nM) in MM1.S cell line for 48 h.

To explore the anti-tumor mechanism of **BC-05**, cell apoptosis and cell cycle assay were performed on the MM1.S cell line. The results are shown in Figure 4. Cell apoptosis results reveal that **BC-05** significantly induces cell apoptosis in a dose-dependent manner. At the concentration of 1, 2, and 4 µM, **BC-05** induces 12.4%, 73.1%, and 88.0% cell apoptosis, respectively. Caspase-3 cleavage is also obvious in the Western bolt with the treatment of **BC-05** at 1, 2, and 4 µM, indicating the occurrence of cellular apoptosis (Figure 4A,B). The cell cycle results demonstrate that **BC-05** blocks MM1.S cells in the Sub-G1 phase (Figure 4C), which is consistent with the apoptosis results. BC-05 significantly upregulates P21^WAF1/Cip1^, which contributes to its cell cycle arrest and anti-proliferative activity.

#### 2.2.5. In Vivo Pharmacokinetic Study of **BC-05**

**Figure 5:**
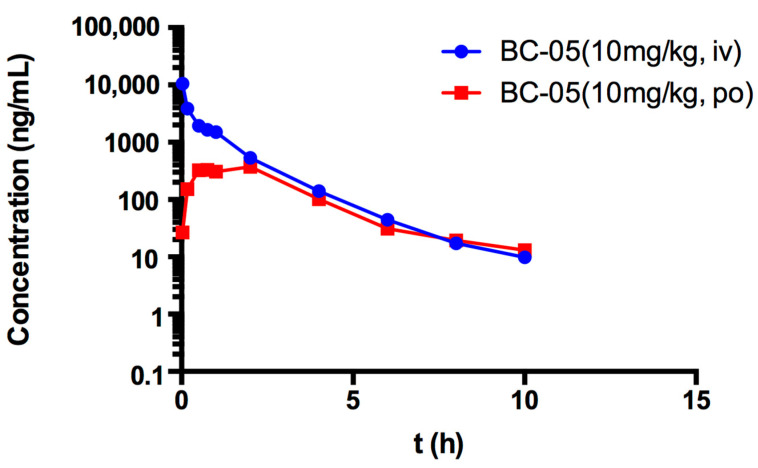
Concentration–time curve after i.v. and oral administration of compound **BC-05** (10 mg/kg) in SD rats (n = 3).

The pharmacokinetic profile of **BC-05** was examined in SD rats. As shown in Table 3 and Figure 5, after a single 10 mg/kg i.v. administration of **BC-05**, the maximum concentration (C_max_), the area under the curve (AUC_0–∞_), half-life (t_1/2_), mean clearance rate (CL) values are 10,488.6 ng/mL, 5154.34 µg/L·h, 1.72 h, and 1.95 L/h/kg, respectively. After the oral administration of compound **BC-05**, at a 10 mg/kg dose, **BC-05** is rapidly absorbed with a time-to-maximum-concentration (T_max_) of 2 h, a C_max_ of 373.2 ng/mL, t_1/2_ of 2.35 h, an AUC_0–∞_ of 1307.7 µg/L·h and a CL of 7.69 L/h/kg. The oral bioavailability (F%) of **BC-05** is 24.9%, which is sufficient to support its oral administration for further in vivo anti-tumor evaluation.

#### 2.2.6. **BC-05** Exhibits Remarkably Anti-Metastasis Efficacy In Vivo

**Figure 6:**
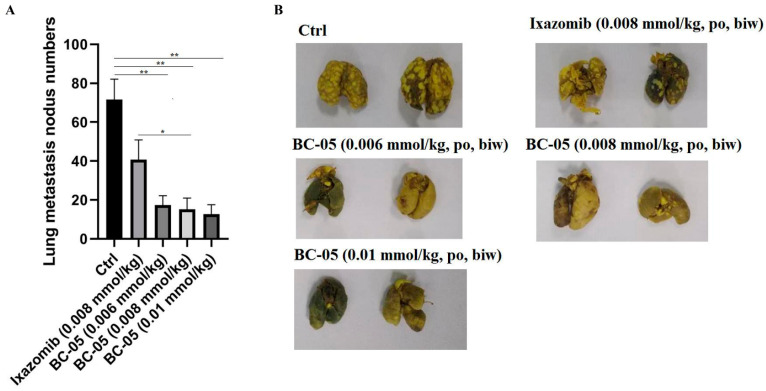
In vivo anti-metastasis activities of **BC-05** in H22 pulmonary metastasis tumor model. (**A**) Lung metastasis nodus numbers in different groups, eight mice per group (* *p* < 0.05; ** *p* < 0.01; Student’s *t*-test). Error bars indicate the standard error of the mean (SEM). (**B**) Representative photographs of lungs.

Cancer metastasis is considered an important reason for treatment failure and postoperative relapse. Our previous work has reported that CD13 inhibitors exhibited potent anti-metastasis activity in vivo [32]. Herein, we conducted the in vivo anti-metastasis study of **BC-05** in a H22 pulmonary metastasis tumor model using ixazomib as a positive control. The mice were randomly divided into five groups and were treated with ixazomib (0.008 mmol/kg, p.o., twice-weekly (BIW)) or **BC-05** (0.006, 0.008, and 0.01 mmol/kg, p.o., BIW) for 2 weeks. The number of H22 pulmonary nodes was calculated at the end of treatment. The results are shown in Figure 6. Compound **BC-05** decreases the number of H22 pulmonary nodes to 17.3, 15.2, and 12.7, respectively, which is much less than that of the vehicle group (71.5). The anti-metastasis activity of ixazomib is much weaker than **BC-05** at the same dose (0.008 mmol/kg).

#### 2.2.7. **BC-05** Exhibits Immunostimulating Potency In Vivo

Frequent use of cytotoxic drugs can damage the body’s immune system. Ubenimex was used as an immunomodulator in cancer treatment and was found to be effective in life prolongation of mice treated with cytotoxic drugs [33,34,35]. Therefore, the carbon clearance index (K) and phagocytosis index (α) representing macrophage phagocytic activity (MPA) of the reticular endothelial system were measured to determine the immunomodulatory effect of compound **BC-05**. According to the data shown in Table 4, the ixazomib-treated group showed lower K and α values than the control group, while **BC-05**-treated groups exhibited higher K and α values than the control group, indicating the immunostimulating potency of **BC-05** in vivo.

#### 2.2.8. **BC-05** Exhibits Superior Anti-Tumor Activity In Vivo

**Figure 7:**
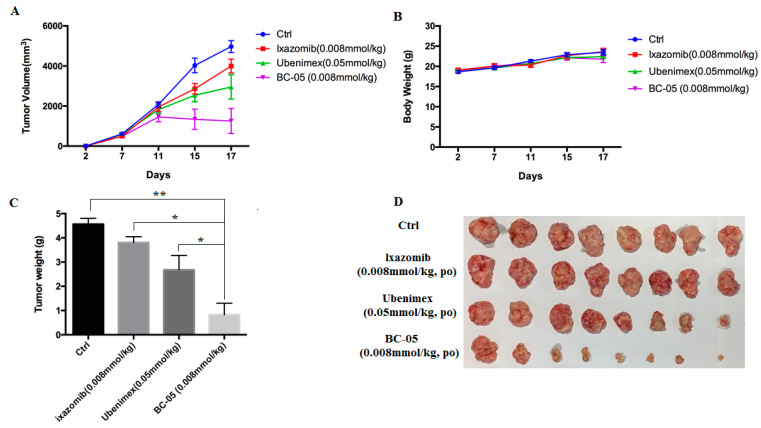
Anti-tumor effect of **BC-05** as tested using tumor-bearing mice. (**A**) Tumor volume or (**C**) tumor weights in different groups treated by oral administration (* *p* ≤ 0.05; ** *p* ≤ 0.01 Student’s *t*-test). (**B**) Body weights in different groups treated by oral administration. (**D**) The photographs of tumors from tumor-bearing mice treated by oral administration. Error bars indicate SEM.

**Figure 8:**
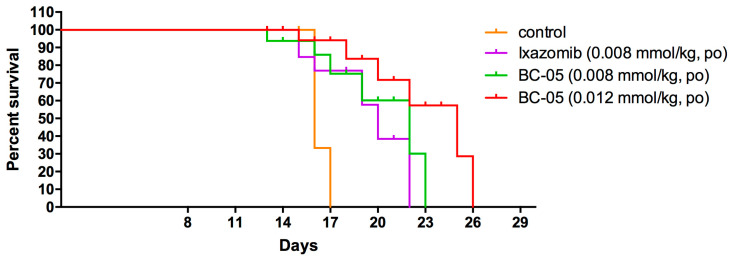
Kaplan–Meier curves showing survival of C57BL/6 mice treated with normal PBS (yellow), ixazomib (purple), and **BC-05** (green and red).

**BC-05** was further evaluated for the in vivo anti-tumor activity using a subcutaneous P3x63Ag8.653 mice model. Ixazomib and ubenimex were used as a positive control. As depicted in Figure 7, treatment with **BC-05** (0.008 mmol/kg) remarkably reduces the tumor volume compared with the control, with T/C of 31.2%. Ubenimex exhibits moderate anti-tumor activity at a dose of 0.05 mmol/kg. The anti-tumor activity of **BC-05** (0.008 mmol/kg) is better than the positive drug ixazomib (0.008 mmol/kg) and ubenimex (0.05 mmol/kg). No obvious body weight loss and discernible side effects are found during the administration of **BC-05**. **BC-05** was further evaluated for the lifespan extension effects in B16F10-bearing C57BL/6 mice (Figure 8). Oral treatment with **BC-05** (0.008 and 0.012 mmol/kg) significantly increases the survival time when compared with the vehicle group. The median survival time (MST) of mice treated with **BC-05** (0.008 mmol/kg), **BC-05** (0.012 mmol/kg), or ixazomib (0.008 mmol/kg) increased to 1.4-fold (22 days), 1.6-fold (25 days), or 1.25-fold (20 days), respectively, compared to the vehicle group (16 days). It should be noted that although ixazomib displays higher anti-proliferative activity than **BC-05** in vitro, it establishes much lower activity in vivo. This may be due to the anti-metastasis (Figure 6) and immunostimulating activity of **BC-05** (Table 4).

#### 2.2.9. Acute Toxicity Assessment of **BC-05** in Healthy Mice

In order to investigate the in vivo safety properties of **BC-05**, we performed a single-dose acute toxicity test of **BC-05** in healthy mice. The mice were randomly divided into seven groups and 10 mice per group (half of males and half of females). **BC-05** at the dose of 0.27 mmol/kg (126.56 mg/kg), 0.36 mmol/kg (168.75 mg/kg), 0.49 mmol/kg (225 mg/kg), 0.65 mmol/kg (300 mg/kg), and 0.86 mmol/kg (400 mg/kg) were administrated orally. The mice were observed for 7 days after treatment of **BC-05**. After oral administration of **BC-05** at 0.27 mmol/kg, no mice died for up to one week (Table 5). The LD_50_ value of **BC-05** is determined to be 0.40 mmol/kg. All the ixazomib-treated mice died at the dose of 0.04 mmol/kg (Table 5). These results suggest that compound **BC-05** shows lower acute toxicity than the approved drug ixazomib. As **BC-05** establishes higher anti-tumor activity than ixazomib at the same concentration (0.008 mmol/kg), it has the potential to be developed as a potential candidate with better efficacy and tolerability profiles.

**Figure 9:**
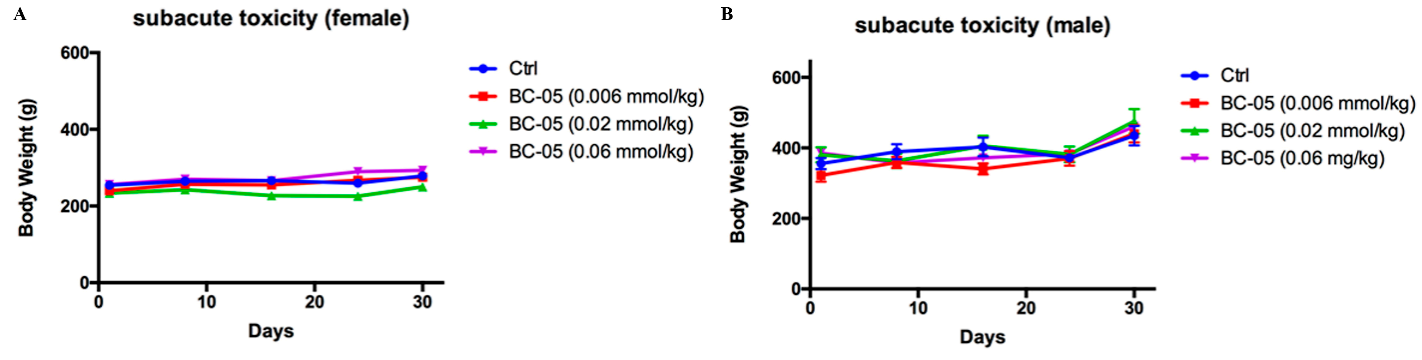
Body weights in male (**A**) or female (**B**) groups treated by oral administration of **BC-05**.

The subacute toxicity of **BC-05** was investigated in SD rats. The SD rats were randomly divided into four groups and were orally administered with **BC-05** twice a week at the doses of 0.006 mmol/kg (2.96 mg/kg), 0.02 mmol/kg (8.87 mg/kg), and 0.06 mmol/kg (26.6 mg/kg) for 30 days. The body weight of the tested animals was monitored throughout the treatment period. After 30 days, no death or behavioral abnormality, such as lethargy, ruffled fur, anorexia, or clonic convulsion, was observed. Moreover, both male and female animals in the tested groups did not exhibit any significant body weight loss during the treatment period (Figure 9).

## 3. Discussion

CD13 is known as a “moonlighting ectoenzyme” and plays a crucial role in tumor development [4]. Despite numerous attempts to design and synthesize CD13 inhibitors, only ubenimex is currently available on the market [3,20]. The development of CD13-targeted drugs primarily relies on multi-targeted drug design strategies and CD13-targeted drug conjugated strategies, such as antibody-drug conjugates (ADC), peptide drug conjugates (PDC), and small-molecule drug conjugates (SMDC) [10,36,37]. In our previous study, we designed and synthesized prodrugs by conjugating ubenimex with cytotoxic drugs, including 5FU and gemcitabine [25,30,31,32]. Upon entering the body, these compounds undergo multiple hydrolysis steps, generating ubenimex and cytotoxic drugs that act in a coordinated synergistic manner. While these conjugates have demonstrated significant anti-tumor activity, designing the linker chain has posed a challenge. An overly stabilized linker chain can reduce in vivo anti-cancer activity, while inadequate stability of the linker chain can result in a short half-life and easy metabolism in vivo [30,32]. To address these issues, we utilized a molecular hybridization strategy to design **BC-05**, a dual-target inhibitor that combines the activities of both CD13 and 20S proteasome inhibitors. Unlike other conjugates, **BC-05** does not require hydrolysis to generate parent drugs to exhibit efficacy against both targets. This presents a novel strategy for developing anti-cancer drugs that are based on CD13.

## 4. Materials and Methods

### 4.1. Chemistry

Unless otherwise stated, all commercial materials, reagents, and solvents were used without further purification. All reactions were assessed by thin layer chromatography (TLC, Yantai, China) with 0.25 mm silica gel plates (GF-254) and visualized by UV light (254 and 365 nm), iodine stain, and ninhydrin. Flash chromatography was performed using the automated CombiFlash R_f_ system (Teledyne ISCO, Lincoln, NE, USA) via 200–300 mesh silica gel. ^1^H NMR and ^13^C NMR spectra were recorded on a Bruker DRX spectrometer at 400 MHz. Using tetramethylsilane as an internal standard, the chemical shifts (*δ*) were represented in parts per million (ppm), and the coupling constants *J* were represented in hertz. High-resolution mass spectrometry (HRMS) was collected at the Marine Biomedical Research Institute of Qingdao, China.

*(2S,3R)-3-(((benzyloxy)carbonyl)amino)-2-hydroxy-4-phenylbutanoic acid* (**2**). AHPA (**1**, 19.50 g, 100.0 mmol) was dissolved in the mixture of THF/H_2_O. Then, the K_2_CO_3_ (41.46 g, 300.0 mmol) and benzyl chloroformate (17.01 g, 100.0 mmol) were added at 0 °C. The mixture was stirred at rt for 6 h. The THF was removed, and the residue was acidified with 1 M HCl to pH 1-3. The EtoAc was added, and the organic layer was collected and washed with brine three times and dried over anhydrous Na_2_SO_4_. The EtoAc was evaporated, and the white solid **2** (25.5 g, yield 77%) was obtained. ^1^H NMR (400 MHz, CD_3_OD) δ 7.32–7.18 (m, 10H), 5.03–4.95 (m, 2H), 4.29 (td, *J*_1_ = 2.40 Hz, *J*_2_ = 6.32 Hz, 1H), 4.09 (d, *J* = 2.40 Hz, 1H), and 2.97–2.77 (m, 2H).

*Benzyl ((2R,3S)-3-hydroxy-4-(((R)-3-methyl-1-((3aS,4S,6S,7aR)-3a,5,5-trimethylhexa-hydro-4,6-methanobenzo[d][1,2,3]dioxaborol-2-yl)butyl)amino)-4-oxo-1-phenylbut-an-2-yl)carbamate* (**4**). The solution of compound **2** (10 g, 30.4 mmol), HOBt (4.51 g, 33.4 mmol), and EDCI (6.4 g, 30.4 mmol) in dry DMF was stirred at 0 °C for 30 min. Then, compound **3** (11.51 g, 30.4 mmol) and DIPEA (4.71 g, 36.4 mmol) in DMF were added. The reaction mixture was stirred at rt for 24 h. After completion, the reaction was quenched with water and extracted with EtoAc. The EtoAc was washed with water, 1N HCl, saturated NaHCO_3,_ and Brine three times and dried over anhydrous Na_2_SO_4_. The EtoAc was removed, and the crude product was purified via column chromatography (DCM/MeOH = 20:1, *v*/*v*) to obtain white solid **4** (12.01 g, yield 68%). ^1^H NMR (400 MHz, CD_3_OD) δ 7.32–7.15 (m, 10H), 5.02–4.92 (m, 2H), 4.38–4.07 (m, 2H), 3.99–3.94 (m, 1H), 3.23–3.10 (m, 1H), 3.01–2.76 (m, 4H), 2.44–2.08 (m, 1H), 2.01–1.53 (m, 4H), 1.46–1.18 (m, 8H), and 1.00–0.77 (m, 9H). 

*((R)-1-((2S,3R)-3-(((benzyloxy)carbonyl)amino)-2-hydroxy-4-phenylbutan-amido)-3-methylbutyl)boronic acid (***5***).* To a solution of compound **4** (10 g, 17.4 mmol), isobutane-boronic acid (3.53 g, 34.7 mmol) in a mixture of methanol and hexane was stirred at 0 °C. A total of 30 mL of 2 mol/L HCl was added, and the mixture was stirred at rt for 3 h. Methanol was removed, and the residue was extracted with DCM (3 × 50 mL). The combined DCM layer was washed with brine three times and dried over anhydrous Na_2_SO_4_. The DCM was removed, and the compound 5 (7.12 g, yield 92%) was obtained by recrystallizing with acetonitrile. ^1^H NMR (400 MHz, CD_3_OD) δ 7.34-7.18 (m, 10H), 5.07–4.92 (m, 2H), 4.26 (d, *J* = 2.36 Hz, 1H), 4.23 (td, *J*_1_ = 2.36 Hz, *J*_2_ = 7.72 Hz, 1H), 2.92–2.83 (m, 2H), 2.67 (t, *J* = 7.60 Hz, 1H), 1.69–1.61 (m, 1H), and 1.35–1.28 (m, 2H).

*2,2′-(2-((R)-1-((2S,3R)-3-(((benzyloxy)carbonyl)amino)-2-hydroxy-4-phenylbutan-amido)-3-methylbutyl)-5-oxo-1,3,2-dioxaborolane-4,4-diyl)diacetic acid* (**6**). To a solution of compound **5** (7 g, 15.83 mmol), citric acid (3.04 g, 15.83 mmol) in EtoAc was stirred at 60 °C for 30 min. The EtoAC was removed, and the residue was washed with DCM. The compound **6** (4.74 g, yield 50%) was obtained by recrystallizing with EtoAC. ^1^H NMR (400 MHz, CD_3_OD) δ 7.33-7.07 (m, 10H), 5.06-4.94 (m, 2H), 4.50-4.39 (m, 1H), 4.29-4.15 (m, 1H), 3.02-2.63 (m, 7H), 1.80-1.62 (m, 1H), 1.36-1.28 (m, 2H), and 0.93-0.88 (m, 6H). 

*2,2′-(2-((R)-1-((2S,3R)-3-amino-2-hydroxy-4-phenylbutanamido)-3-methylbutyl)-5-oxo-1,3,2-dioxaborolane-4,4-diyl)diacetic acid* (**7**). Compound **6** (10 g, 16.7 mmol) was dissolved in 50 mL dry methanol, and the palladium-carbon (1.5 g) was added, and the reaction was stirred under a hydrogen atmosphere for 16 h. After completion, the palladium carbon was filtered, and the methanol was evaporated. Compound **7** (6.20 g, yield 80%) was obtained by washing with ethyl ether. ^1^H NMR (400 MHz, CD_3_OD) δ 7.37–7.23 (m, 5H), 4.18–4.10 (m, 1H), 3.88–3.84 (m, 1H), 3.28–3.10 (m, 1H), 3.02–2.49 (m, 6H), 1.76–1.64 (m, 1H), 1.46–1.28 (m, 2H), 0.94–0.86 (m, 6H), 0.93–0.88 (m, 6H). ^13^C NMR (101 MHz, CD_3_OD) δ 180.80, 172.63, 172.26, 172.00, 136.16, 129.31, 128.68, 128.53, 127.09, 126.88, 77.25, 70.15, 54.76, 43.49, 42.12, 41.72, 35.49, 24.97, 23.13, and 20.99. HRMS (AP-ESI) *m*/*z* calculated for C_21_H_29_BN_2_O_9_ [M − H]^−^ 463.18934, found 463.18607.

### 4.2. CD13 Inhibition Assay [20]

*L*-leucine-*p*-nitroanilide can be degraded by recombinant human aminopeptidase (R&D systems) or procine aminopeptidase (Sigma Chemical Co., St. Louis, MO, USA) and produce light absorption at 405 nm. The CD13 inhibitory activity of synthetic compounds was identified according to the degree of degradation of *L*-leucine-*p*-nitroanilide. *L*-leucine-*p*-nitroanilide was dissolved in DMSO and mixed with tested compounds in a transparent 96-well plate; then, aminopeptidase was added using phosphate solution (pH 7.2) as the assay buffer. After incubation at 37 °C for 30 min, the absorption was detected under a 405 nm wavelength via Tecan Spark multimode microplate. 

### 4.3. 20S Proteasome Inhibition Assay

The 20S proteasome inhibition assay was performed using the Proteasome-Glo™ Chymotrypsin-Like Assay kit (Promega, Alexandria, NSW, Australia). Suc-LLVY-aminoluciferin is a substate that can be cleaved by the 20S proteasome, release the luciferase (aminoluciferin) and allow the luciferase reaction to produce light. Gradient concentration of tested compounds and the purified 20S proteasome enzyme (R&D systems, Minneapolis, MN, USA) were successively added into a transparent 96-well plate and mixed thoroughly. Then, 50 µL of a Suc-LLVY-AMC (10 μM) reagent was added. Gently mix contents with a plate shaker at 300 to 500 rpm for 30 s. Incubate at room temperature for an hour. The luminescence was recorded via the Tecan Spark multimode microplate.

### 4.4. Molecular Docking

The molecular docking process was performed using Glide (Schrodinger Inc., supported by the Shanghai Institute of Materia Medica Chinese Academy of Sciences). Crystal structures of CD13 (PDB: 2DQM) and 20S proteasome (PDB: 7LXT) were chosen as receptors in the docking studies. Structural modifications were performed to make the proteins suitable for the docking. The water molecules and the ligand crystallized in the protein structure were removed. The OPLS 2005 force field was assigned to the refined structure. The structure of **BC-05** was sketched by Maestro and prepared by LigPrep. The active site of CD13 was defined as a cubic box at a distance of 20 Å, and that of 20S protease is also similar. The active site of 20S protease was applied with extra precision in the docking process, and other parameters were set as default.

### 4.5. Cell Proliferation Analysis

All tumor cell lines in the current research were grown in a certain growth medium containing 10% FBS and 1% (*v*/*v*/*v*) penicillin–streptomycin–amphotericin at 37 °C in 5% CO_2_. Cells were seeded into 96-well plates at a density of 8 × 10^3^ cells per well for tumor adherent cells and 2 × 10^4^ cells per well for tumor-suspended cells in a final volume of 100 μL. After 12 h, 100 μL of various concentrations of compounds was added to each and incubated for 48 h. A 0.5% MTT solution was added per well before the intermixture was incubated for another 4 h, and formazan formed from MTT was extracted by adding 150 mL of DMSO rocking for 15 min. Absorbance was measured at 570 nm using a Spark multimode microplate from Tecan. The IC_50_ values were calculated according to the inhibition ratios. 

### 4.6. Cell Cycle and Apoptosis Study

For the cell cycle assay, MM1.S cells were plated in 6-well plates with a density of 2.5 × 10^6^ cells per well. A total of 12 h later, **BC-05** (1, 2, and 4 μM), ixazomib (10 nM), or a vehicle (0.5% DMSO) was added, and the mixture was incubated for 48 h. Subsequently, cells were harvested, washed with cold PBS, and fixed in 75% cold ethanol at −20 °C overnight. After washing off the ethanol, fixed cells were treated with RNase A, dyed with propidium iodide solution (20 μg/mL), and incubated in the dark at 37 °C for 30 min. The final assay was analyzed using a Beckman flow cytometer.

For the cell apoptosis assay, MM1.S cells were plated in 12-well plates with a density of 6 × 10^5^ cells per well and treated with **BC-05** (1, 2, and 4 μM), ixazomib (10 nM) or a vehicle (0.5% DMSO) for 48 h after 12 h fixation. Then, cells were harvested, washed with a cold PBS buffer, and resuspended in 500 μL of an annexin V binding buffer. After being stained with 5 μL of Annexin V-FITC and 5 μL of propidium iodide, the cells were detected by a flow cytometer (Beckman Coulter Inc., Sharon Hill, PA, USA).

### 4.7. Western Blot Analysis

MM1.S cells were seeded in 6-well plates with a density of 2.5 × 10^6^ cells/well. Then, the cells were cultured with 1, 2, and 4 μM **BC-05** and 10 nM ixazomib, respectively. After incubation for 24 h, the cells were collected, and total proteins were extracted via RIPA containing a 2% protease inhibitor cocktail. Cell lysates were centrifugated at 4 °C, 12,000 rpm for 15 min, and the supernatants were collected and quantified with BCA assay. After being boiled at 100 °C for 10 min in a NuPAGE lithium dodecyl sulfate (LDS) sample buffer (5X), equivalent samples were separated by 12% sodium dodecyl-sulfate polyacrylamide gel electrophoresis at 120 V, transferred to immune-blot poly(vinylidene fluoride) (PVDF) membranes (Merck Millipore, Burlington, MA, USA) at 4 °C and then blocked by 5% non-fat milk in TBST for an hour. The membranes were incubated overnight at 4 °C with certain primary antibodies, which were deliquates by an antibody dilution buffer (Boster Bio, Pleasanton, CA, USA) and then washed with TBST. After incubating with a horseradish (HRP)-conjugated secondary antibody for 1 h at room temperature, the membranes were washed five times with TBST for 5 min. The images were visualized on the Tanon-5200 Chemiluminescent Imaging System via luminol reaction. The primary antibodies pro-caspase-3 (ab2302), cleaved caspase-3 (ab32150), P21 (sc-6246), and GAPDH (sc-32233) were purchased from Abcam, Abcam, and Santa Cruz Biotechnology, respectively. 

### 4.8. In Vivo Pharmacokinetics

The animal experiments were conducted under the guidelines of the “Regulations for the Administration of Affairs Concerning Experimental Animals” (revised 2017, State Council No. 676). The pharmacokinetic properties (PK) were performed in SD rats, and the procedures conducted were in accordance with institutional guidelines. Male SD rats (weighting 200 ± 20 g) fasted for 12 h and were divided into two groups, three in each group: oral administration of **BC-05** (10 mg/kg) and intravenous administration of **BC-05** (10 mg/kg). A total of 300 μL of a blood sample was collected through retro-orbital plexus from the rats in each group at 0 h, 0.033 h, 0.167 h, 0.5 h, 0.75 h, l h, 2 h, 4 h, 6 h, 8 h, 10 h, and 24 h. Plasma samples were placed in microcentrifuge tubes containing Li-heparin to avoid coagulant, separated by centrifugation at 12,000 rpm for 10 min, and stored at −80 °C until sample analysis. After the precipitation of proteins, samples were centrifuged at 15,000 rpm for 10 min, and the supernatant was reserved for HPLC-MS/MS analysis. 

### 4.9. In Vivo H22 Pulmonary Metastasis Model

The animal experiments were conducted under the guidelines of the “Regulations for the Administration of Affairs Concerning Experimental Animals” (revised 2017, State Council No. 676). H22 cells (3 × 10^6^ cells/100 μL per mouse) were intravenously (i.v.) inoculated via the tail vein of Kunming mice aged 6 weeks on day 0. To ensure the growth of pulmonary metastasis before the drug treatment, the tumor was allowed to develop for 7 days. On day 8, the randomly grouped mice were treated with ixazomib (0.008 mmol/kg, p.o., BIW) or **BC-05** (0.006, 0.008, and 0.01 mmol/kg, p.o., BIW) for consecutive 2 weeks. On day 15, the mice were weighed and sacrificed, and the lungs were removed and weighed. After being fixed in the Bouin stationary solution for one day, the lung metastasized nodes of each mouse were numbered.

### 4.10. In Vivo P3x63Ag8.653 Tumor Transplant Models

The animal experiments were conducted under the guidelines of the “Regulations for the Administration of Affairs Concerning Experimental Animals” (revised 2017, State Council No. 676). For the P3x63Ag8.653 tumor development, male mice were subcutaneously injected with 200 μL murine P3x63Ag8.653 cells with a density of 1 × 10^7^/mL. On the eighth day after injection, the transplanted mice were randomized into treatment and control groups. The tested compounds were administrated orally at the prescribed dose biweekly for two weeks. After two weeks of administration, mice were executed, and the tumor tissues were dissected and weighed. T/C was calculated according to the following formula: T/C = the mean RTV of the treated group/the mean RTV of the control group, where RTV is the relative tumor volume = V_t_/V_0_ (V_t_, the tumor volume measured at the end of treatment; V_0_, the tumor volume measured at the beginning of treatment).

### 4.11. Survival Time in Mice Bearing B16F10 Cells

The animal experiments were conducted under the guidelines of the “Regulations for the Administration of Affairs Concerning Experimental Animals” (revised 2017, State Council No. 676). Female C57/B6 mice at 5 to 6 weeks were implanted via intraperitoneal (ip) injection with 5.0 × 10^6^ B16F10 cells per individual and randomly assigned into control groups and treatment groups. Seven days after inoculation, mice from the control group were treated orally with DMSO in a vehicle, whereas the compound group mice were treated with specified concentrations of compounds by oral administration twice a week for two weeks. The survival percentage of the mice in each group was recorded and plotted. The anti-tumor activity of the drug was evaluated by measuring the median survival time (MST) for each group. 

### 4.12. Macrophage Phagocytosis by Carbon Clearance Test

The animal experiments were conducted under the guidelines of the “Regulations for the Administration of Affairs Concerning Experimental Animals” (revised 2017, State Council No. 676). Kunming mice were randomly divided into control groups and treatment groups. After oral administration of test compounds twice a week for two weeks, the mice were injected with carbon ink suspension (Indian Ink, Camel) (100 μL/10 g of body weight) via the tail vein. Two and ten minutes after the injection of carbon suspension, the blood samples were collected from a retro-orbital vein. A total of 25 μL of a blood sample was directly added into 2 mL of 0.1% sodium carbonate solution. The absorbance of the sample was measured at 600 nm using a UV-visible spectrophotometer. The K value was calculated by the formula Log (OD_2_) − Log (OD_10_)/(T_2_ − T_1_), where OD_2_ and OD_10_ were the absorbance of blood at 2 and 10 min, respectively; T_2_ is the last time point of blood collection; T_1_ is the first time point of blood collection. The α value was calculated by the formula (k^1/3^ × body weight)/(liver weight + spleen weight).

### 4.13. Acute Toxicity Experiment

The animal experiments were conducted under the guidelines of the “Regulations for the Administration of Affairs Concerning Experimental Animals” (revised 2017, State Council No. 676). Kunming mice (10−20 g) were fasted for 12 h and randomly divided into five groups (10 mice per group with half of males and half of females). Groups of mice were administrated orally with **BC-05** at a dose of 0.27 mmol/kg (126.56 mg/kg), 0.36 mmol/kg (168.75 mg/kg), 0.49 mmol/kg (225 mg/kg), 0.65 mmol/kg (300 mg/kg), and 0.86 mmol/kg (400 mg/kg) or ixazomib at a dose of 0.04 mmol/kg (20 mg/kg), respectively. The mouse mortality was monitored daily and recorded up to 7 days after treatment. 

### 4.14. Subacute Toxicity Experiment

The animal experiments were conducted under the guidelines of the “Regulations for the Administration of Affairs Concerning Experimental Animals” (revised 2017, State Council No. 676). For the subacute toxicity study, 12 male and 12 female SD rats (purchased from the Weifang Medical University) were randomly divided into three treatment groups and a control group (n = 6, half male and half female). The rats in treatment groups were given 0.06 mmol/kg (p.o. BIW), 0.02 mmol/kg (p.o. BIW), and 0.006 mmol/kg (p.o. BIW) **BC-05,** respectively, for 30 days, while the rats in control groups were treated with the same volume of a vehicle solution. The rats were weighed each day and dissected at day 30. Behavioral abnormalities such as lethargy, ruffled fur, anorexia, clonic convulsion, and diarrhea were recorded throughout the entire experimental period.

### 4.15. Statistical Analysis

The statistical significance of differences between the groups was assessed by Student’s *t*-test. *p*-values < 0.05 were considered statistically significant. 

## 5. Conclusions

In summary, we first designed, synthesized, and evaluated the CD13/proteasome dual inhibitor **BC-05**. **BC-05** is 7.5–14.6 times more potent than ubenimex in porcine and human CD13 enzymatic inhibitory activity and exhibits moderate inhibitory activity toward the 20S proteasome. The in vitro cell-based study reveals that **BC-05** displays moderate anti-proliferation activity, which is better than the positive control ubenimex and less potent than ixazomib. Even though **BC-05** is much more potent than ixazomib in the in vivo anti-tumor study, it has significance in reducing the tumor volume and prolonging life span. The remarkable anti-metastasis and immunostimulating activity of **BC-05** due to CD13 inhibition may contribute to its superior in vivo efficacy. The PK study shows that **BC-05** is a promising oral effective anti-tumor agent with oral F% of 24.9%. Furthermore, acute and subacute toxicity studies demonstrated that **BC-05** is much less toxic than ixazomib. Overall, these in vitro and in vivo results illustrate that compound **BC-05** is a promising candidate for the treatment of multiple myeloma. 

## Figures and Tables

**Table 1:** CD13 and 20S proteasome inhibitory activity of compound **BC-05**.

Cpd.	IC_50_(μM) *^a^* toward CD13 from Porcine Kidney	IC_50_(μM) *^a^* toward Human CD13	IC_50_(μM) *^b^* toward Chymotrypsin-like (CT-L)
**BC-05**	0.65 ± 0.05	0.13 ± 0.01	1.39
Ixazomib	>20	N.D.	0.0024
Ubenimex	4.91 ± 0.13	2.03 ± 0.18	>10

*^a^* Assays were performed in replicate (n ≥ 3). Data are shown as mean ± standard deviation (SD); *^b^* assays were performed in replicate (n ≥ 3); the SD values are <20% of the mean. “N.D.” means not detected.

**Table 2:** Anti-proliferative activities of **BC-05** against selected cancer cell lines and non-malignant cell lines with ixazomib and ubenimex as a positive control (IC_50_ in μM *^a^*).

Cell Lines	BC-05	Ixazomib	Ubenimex
U266	13.66 ± 1.43	0.12 ± 0.01	>200
RPMI-8266	15.12 ± 2.23	0.098 ± 0.01	>200
ARP-1	10.23 ± 1.23	0.099 ± 0.002	>200
MM.1S	1.53 ± 0.24	0.007 ± 0.002	>200
HEL	13.26 ± 2.81	0.11 ± 0.003	>200
KG1	41.55 ± 4.11	0.40 ± 0.05	>200
P3x63Ag8.653	17.73 ± 0.75	0.08 ± 0.005	>200
SP2	14.67 ± 4.91	0.074 ± 0.017	>200
A2780	33.71 ± 0.99	0.11 ± 0.01	>200
HCT116	86.66 ± 7.34	0.02 ± 0.01	>200
Hela	83.11 ± 8.79	0.74 ± 0.19	>200
MDA-MB-231	13.30 ± 0.49	0.12 ± 0.01	>200
HT-29	12.51 ± 1.21	0.06 ± 0.001	>200
U251	47.54 ± 11.89	0.92 ± 0.86	>200
PC3	47.41 ± 4.22	0.75 ± 0.12	>200
HEK293	>200	17.10 ± 2.27	>200
HL-7702	>200	4.04 ± 0.63	>200

*^a^* Assays were performed in replicate (n ≥ 3); data are shown as mean ± SD.

**Table 3:** Pharmacokinetic parameters of **BC-05** with i.v. and oral administration (n = 3) *^a^*.

	BC-05	BC-05
Administered dose (mg/kg)	i.v. at 10 mg/kg	oral at 10 mg/kg
AUC_0–∞_ (μg/L∗h)	5154.34 ± 482.30	1307.71 ± 116.74
t_1/2_ (h)	1.72 ± 0.67	2.35 ± 1.23
CL (L/h/kg)	1.95 ± 0.18	7.69 ± 0.66
T_max_ (h)	0.03 ± 0	2.0 ± 0
C_max_ (ng/mL)	10,488.6 ± 1414.99	373.2 ± 49.65
F (%)	--	24.9 ± 4.32

*^a^* AUC_0–∞,_ area under curve; CL, mean clearance rate; t_1/2_, half-life; T_max_, time-to-maximum-concentration; C_max_, maximum concentration; F, oral bioavailability. Data are shown as the mean of three rats ± SD.

**Table 4:** The effects of ixazomib and **BC-05** on carbon clearance index K and phagocytosis index α in Kunming mice.

Groups	Dosage (mmol/kg)	Carbon Clearance Index (K) *^a^*	Phagocytosis Index (α) *^a^*
Ctrl	0	0.057 ± 0.0061	5.36 ± 0.19
Ixazomib	0.008	0.045 ± 0.0092	5.00 ± 0.34
**BC-05**	0.006	0.066 ± 0.0077	6.60 ± 0.26
**BC-05**	0.008	0.076 ± 0.0016	6.61 ± 0.05

*^a^* Data are shown as mean ± SD.

**Table 5:** Survival rate of single oral administration of **BC-05** and ixazomib in mice (n = 10).

Groups	Survival Animals
Vehicle	10/10
**BC-05** (0.27 mmol/kg)	10/10
**BC-05** (0.36 mmol/kg)	6/10
**BC-05** (0.49 mmol/kg)	4/10
**BC-05** (0.65 mmol/kg)	1/10
**BC-05** (0.86 mmol/kg)	0/10
Ixazomib (0.04 mmol/kg)	0/10

## Data Availability

Data are contained within the article and Supplementary Materials.

## Supplementary Materials

The following supporting information can be downloaded at https://www.mdpi.com/article/10.3390/molecules28176343/s1, Figure S1: IC_50_ curves of compounds **BC-05**, ubenimex, and ixazomib for CD13. Figure S2: IC_50_ curves of compound **BC-05** and ixazomib for the 20S proteasome. Figure S3: IC_50_ curves of compounds **BC-05**, ubenimex, and ixazomib for cancer cell lines or normal cell lines. Figure S4: ^1^H-NMR, ^13^C-NMR, and HRMS spectral information of target compound **BC-05**.
