# Supplementary material for: Discovery of a Novel Ubenimex Derivative as a First-in-Class Dual CD13/Proteasome Inhibitor for the Treatment of Cancer
Source: Molecules. 2023 Aug 30;28(17):6343. doi: 10.3390/molecules28176343 (PMC10489073; doi:10.3390/molecules28176343)
Supplement: Supplementary file 1 [file molecules-28-06343-s001.zip › molecules-2539965-supplementary.pdf]

Discovery of a Novel Ubenimex Derivative as First-In-Class Dual CD13/Proteasome Inhibitor for the Treatment of Cancer

Jian Zhang[1,†,*], Simin Sun[2,†], Jinyu Liu[2], Liang Zhang[2], Di Guo[1], Naixin Zhang[3], Jun Zhao[4], Dexin Kong[3], Tongqiang Xu[4], Xuejian Wang[1], Wenfang Xu[4,5], Xiaoyang Li[2,4,5], Yuqi Jiang[2,4,*]

[1] College of Pharmacy, Weifang Medical University, 261053 Wei'fang, Shandong, P.R. China.

[2] Key Laboratory of Marine Drugs, Chinese Ministry of Education, School of Medicine and Pharmacy, Ocean University of China, 5 Yushan Road, Qingdao 266003, China.

[3] Tianjin Key Laboratory on Technologies Enabling Development of Clinical Therapeutics and Diagnostics, School of Pharmacy, Tianjin Medical University, Tianjin 300070, China.

[4] Marine Biomedical Research Institute of Qingdao, Qingdao, Shandong, 266071, P.R. China.

[5] Laboratory for Marine Drugs and Bioproducts, Qingdao National Laboratory for Marine Science and Technology, Qingdao, 266237, P.R. China.

* Corresponding Author. J.Z. E-mail: zhangjian_3323@163.com; Y.J. E-mail: jiangyuqi@ouc.edu.cn.

† These authors contributed equally to this work.

Supplementary Figures:

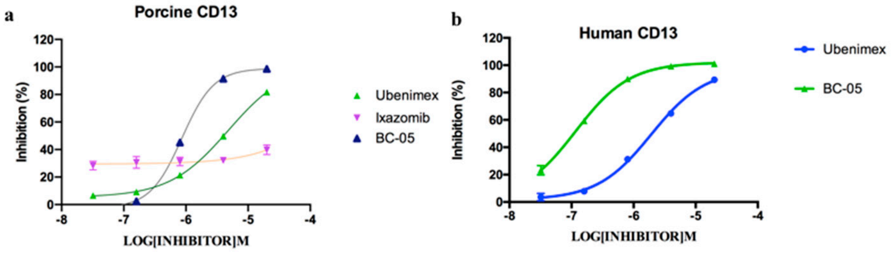

Figure S1. IC_{50} curves of compounds **BC-05**, ixazomib and ubenimex for porcine or human CD13.

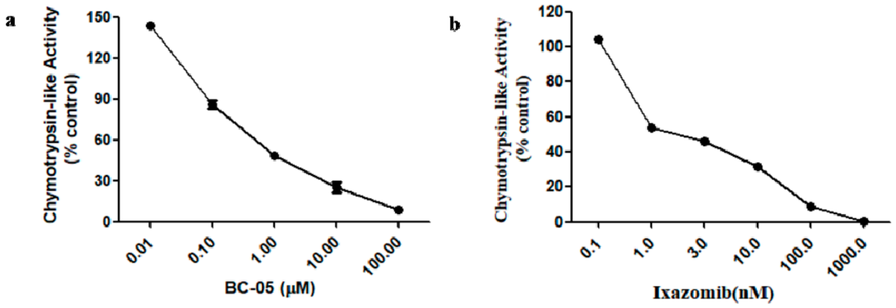

Figure S2. IC_{50} curves of compounds **BC-05** and ixazomib for 20S proteasome.

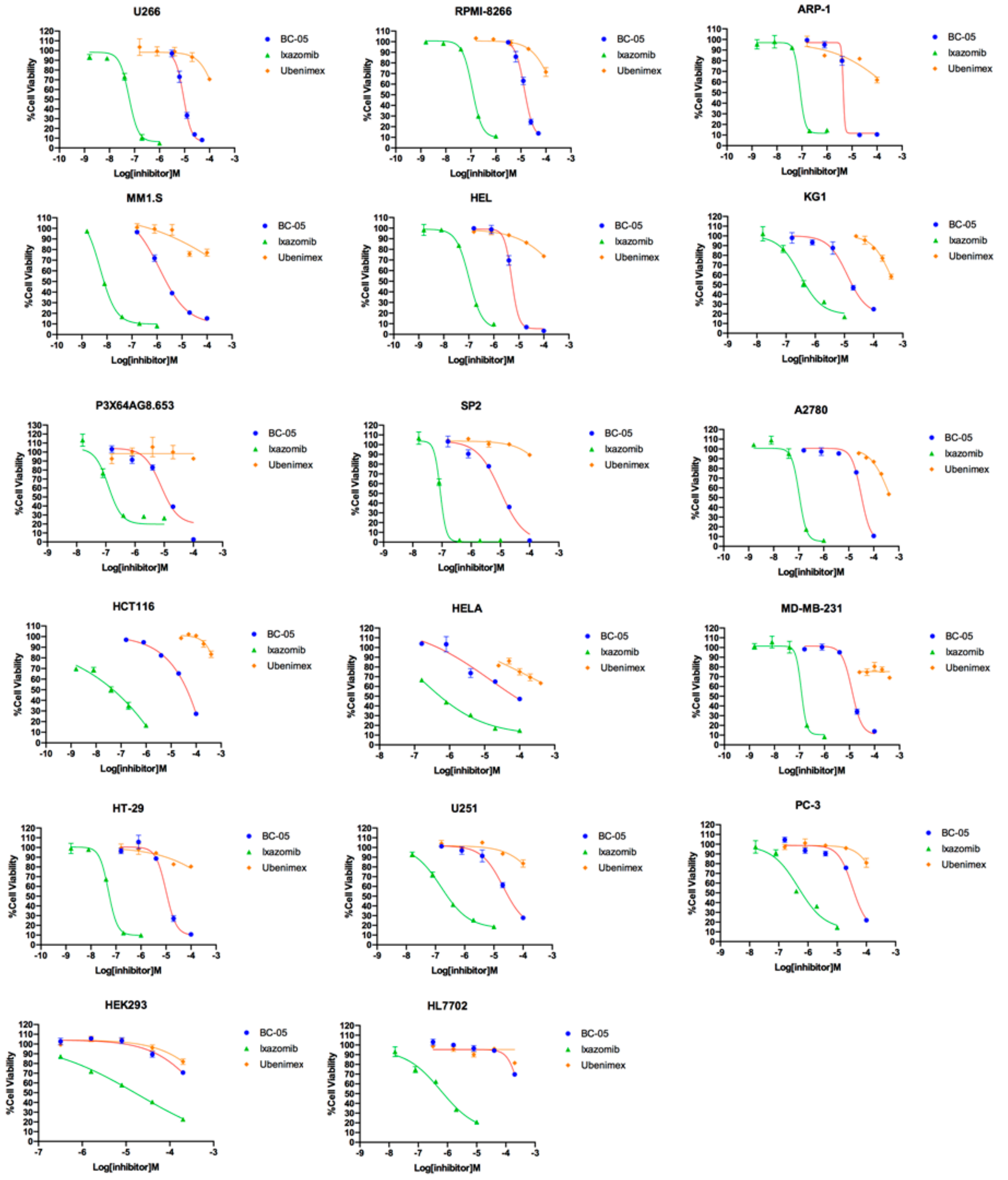

Figure S3. IC_{50} curves of compounds **BC-05**, ixazomib and ubenimex for cancer cell lines or normal cell lines.

(a) ^{1}H NMR spectrum for **BC-05**:

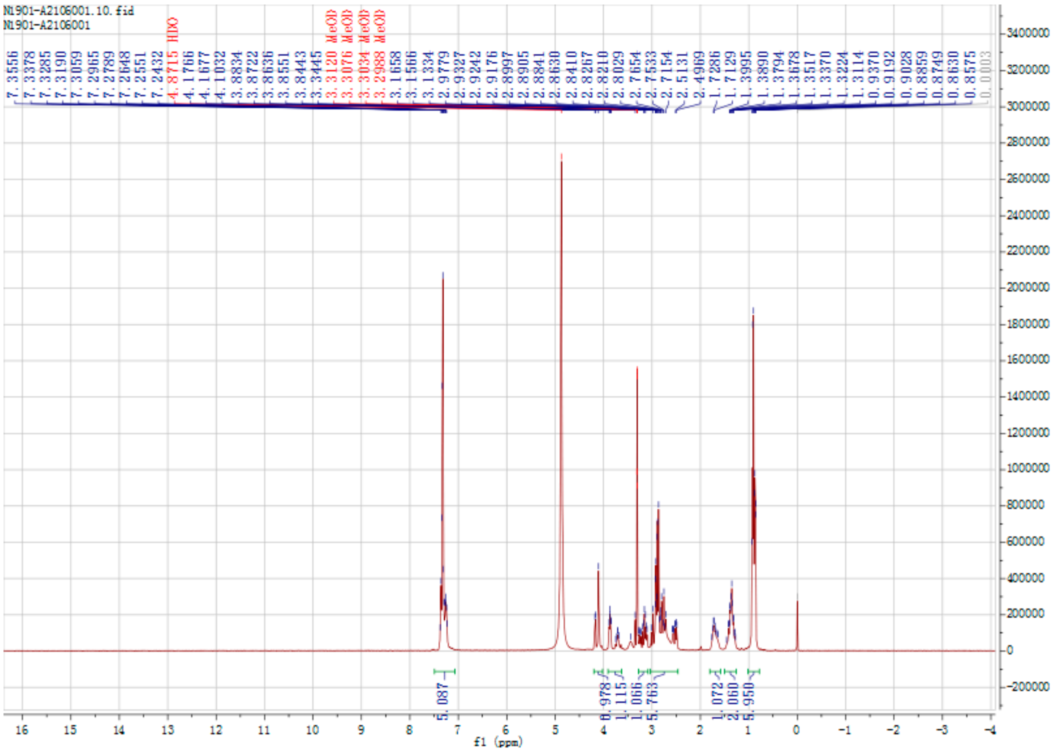

(a) ^{13}C NMR spectrum for **BC-05**:

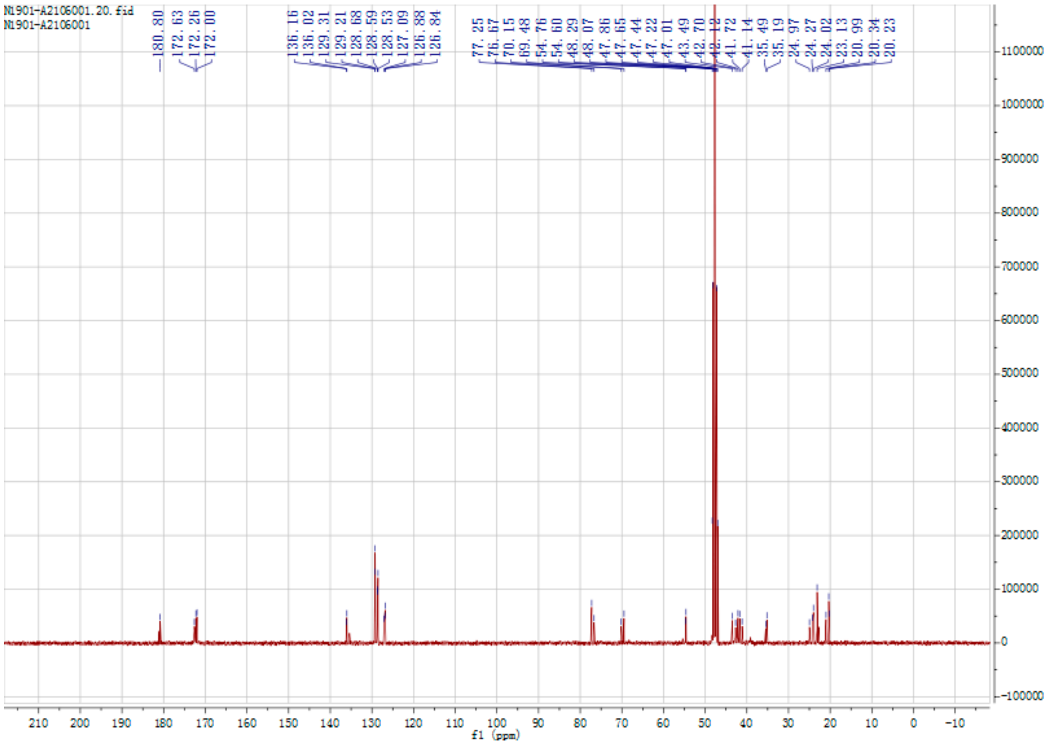

(c) HRMS spectrum for **BC-05**:

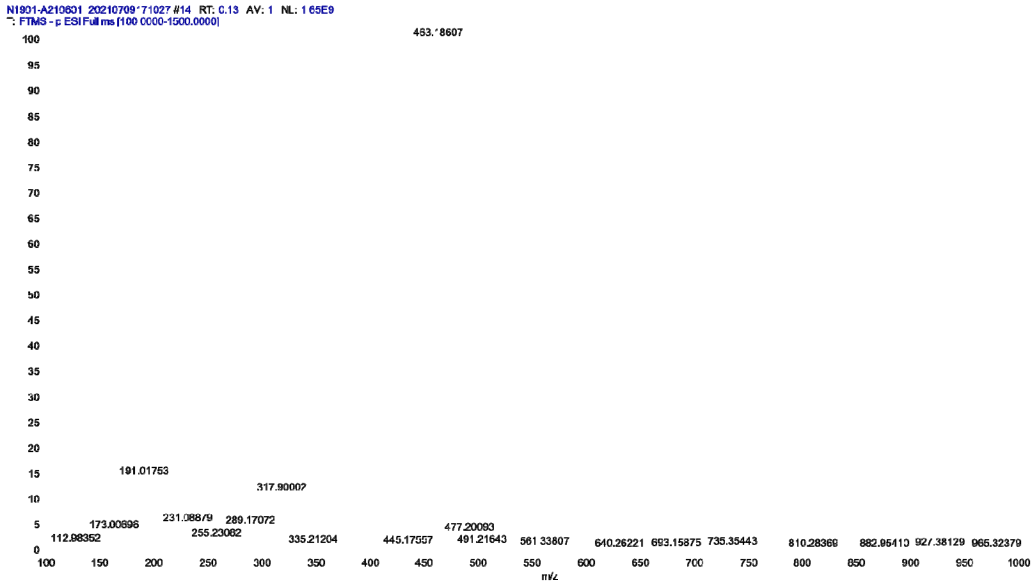

Figure S4. ^{1}H-NMR (a), ^{13}C-NMR (b) and HRMS (c) of **BC-05**.
